# Babao Dan Reverses Multiple-Drug Resistance in Gastric Cancer Cells via Triggering Apoptosis and Autophagy and Inhibiting PI3K/AKT/mTOR Signaling

**DOI:** 10.1155/2021/5631942

**Published:** 2021-07-09

**Authors:** Jinyan Zhao, Weilan Lan, Jun Peng, Bin Guan, Jie Liu, Min Zhang, Zhixue Zhan, Jiumao Lin

**Affiliations:** ^1^Academy of Integrative Medicine, Fujian University of Traditional Chinese Medicine, Fuzhou, Fujian 350122, China; ^2^Fujian Key Laboratory of Integrative Medicine on Geriatric, Fujian University of Traditional Chinese Medicine, Fuzhou, Fujian 350122, China; ^3^Key Laboratory of Integrative Medicine of Fujian Province University, Fujian University of Traditional Chinese Medicine, Fuzhou, Fujian 350122, China; ^4^Xiamen Traditional Chinese Medicine Co., Ltd., Xiamen 361100, China

## Abstract

Multidrug resistance (MDR) is a critical reason for cancer chemotherapy failure. Babaodan (BBD) is a famous traditional Chinese patent medicine reported to have antigastric cancer activity. However, the roles and molecular mechanisms of the reversal of MDR of gastric cancer by BBD have not been well described until now. Therefore, the purpose of this study was to elucidate further the role of BBD in reversing the MDR of gastric cancer cells and its specific regulatory mechanism via *in vitro* experiments. To verify our results, MTT, Doxorubicin (DOX) staining, Rhodamin123 (Rho123) staining, DAPI staining, Annexin V-FITC, propidium iodide (PI), Cyto-ID, and western blot assays were performed. To determine whether BBD triggers apoptosis and autophagy through the PI3K/AKT/mTOR signaling, we also applied 3-methyladenine (3-MA), chloroquine (CQ), and 740Y-P (an activator of PI3K). The results showed that BBD reversed the MDR and induced apoptosis and autophagy of SGC7901/DDP cells. Pathway analyses suggested BBD inhibits PI3K/AKT/mTOR pathway activity and subsequent apoptosis-autophagy induction. Inhibition of autophagy with 3-MA and chloroquine (CQ) was performed to confirm that BBD promoted autophagy. PI3K agonist, 740Y-P, further verified BBD inhibition of PI3K/AKT/mTOR pathway activation. In conclusion, BBD may reverse the MDR of gastric cancer cells, induce apoptosis, and promote autophagy via inactivation of the PI3K/AKT/mTOR signaling pathway.

## 1. Introduction

Gastric cancer (GC) is the third most common cancer worldwide and is associated with high mortality [1, 2]. Gastric cancer incidence and mortality are the highest in East Asia, especially in China [3–5]. The occurrence of stomach cancer is multifactorial, influenced by environmental bacteria, host responses, genetic and epigenetic molecular changes, *Helicobacter pylori* infection, among other factors [6, 7]. The current primary treatment for gastric cancer is surgical resection; however, some advanced or relapsed GC patients are not candidates for surgical treatment, so chemotherapy serves as a lead alternative therapy, especially for advanced gastric cancer [3]. Although medical advances in gastric cancer have improved, patients' predictive and overall survival rates remain poor due to their limited and complex response to chemotherapy [5]. In addition, multidrug resistance is the leading cause of low survival time, which leads to the failure of chemotherapy for tumors and limited efficacy in most patients with gastric cancer. Therefore, how to reverse multidrug resistance remains a major clinical challenge.

Multidrug resistance (MDR) involves different mechanisms that induce cancer cells to develop cross-resistance to various structurally and mechanically unrelated chemotherapy agents, thereby limiting the long-term effective use of chemotherapy drugs [8]. One mechanism mediating MDR is through the regulation of cancer cell apoptosis and autophagy by anticancer medications [9]. Another critical mechanism involves the increased outflow of drugs from cancer cells by upregulated transport proteins that rely on specific energy sources such as ABCB1, ABCC1, and ABCG2 [10]. Other mechanisms that may contribute to drug resistance include EMT, DNA damage repair, drug target mutations, and stem cell modification [11, 12].

Babaodan (BBD) has a long medical history originating from palace secret recipes. It consists of Moschus, natural Calculus Bovis, snake gall, pearl, antelope horn, radix notoginseng, and other precious Chinese medicinal materials. Such components act through clearing away wetness-heat, promoting circulation, detoxification, and pain relief. Many clinical and experimental studies have demonstrated significant antitumor activity by BBD. In addition, these studies highlighted BBD's promising clinical efficacy during posttumor chemotherapy and use as adjuvant therapy for various cancers, including GC [13–16]. Preliminary experimental results corresponding to clinical studies suggest that BBD could significantly inhibit gastric cancer cell proliferation and metastasis and induce their apoptosis [13, 16]. However, the specific regulatory mechanisms by which BBD reverses MDR have not been well studied. Therefore, the purpose of this study was to further elucidate the role of BBD in reversing gastric cancer cell MDR and its specific regulatory mechanism through *in vitro* experiments to provide an experimental basis for the clinical treatment of patients with gastric cancer drug resistance.

## 2. Materials and Methods

### 2.1. Reagents

Babaodan (Chinese FDA approval No. Z10940005) was provided by Xiamen Traditional Chinese Medicine Co., Ltd., China. Fetal bovine serum (FBS) and RPMI-1640 medium were purchased from ThermoFisher Scientific, Inc. (Waltham, MA, USA). Cisplatinum (DDP, 100 mg, MKG2946) was purchased from Millipore-Sigma (MA, USA). FITC-Annexin V/propidium iodide (PI) apoptosis assay kit (cat. no. KGA108) was purchased from Nanjing KeyGene Biotech Co., Ltd. (Nanjing, China). Antibodies to Bax (cat. no. 50599-2), Bcl-2 (cat. no. 12789-1), caspase-3 (cat. no.19677-1), LC3 (cat. no.14600-1-AP), p62 (cat no. 18420-1AP), Beclin 1 (cat. no. 11306-1-AP), PI3K (cat. no. 60225-1), AKT (cat. no. 60203-2), p-AKT (cat. no. 66444-1), and GAPDH (cat no. 60004) were purchased from Proteintech (Chicago, IL, USA). ABCB1 (cat no. 13978), ABCC1 (cat no. 72202), ABCG2 (cat. no. 42078), cleaved caspase-3 (cat. no. 9664), mammalian target of rapamycin (mTOR) (cat no. 2983), and phospho-mTOR (cat no. 5536) were purchased from Cell Signaling Technology, Inc. (Danvers, MA, USA). The enhanced chemiluminescent (ECL) substrate for horseradish peroxidase (HRP) activity was acquired from Proteintech (Chicago, IL, USA). Other reagents, if not explicitly mentioned, were obtained from Nest (Wuxi, China).

BBD (No.180901) was purchased from Xiamen Traditional Chinese Medicine Factory Co., Ltd. (Xiamen, China). The BBD micropowder was diluted in PBS and further diluted with RPMI-1640 medium to different concentrations (0, 0.25, 0.5, and 1.0 mg/ml) prior to use.

### 2.2. Cell Lines and Culture

The human gastric carcinoma parental cell line SGC7901 and cells resistant to DDP (SGC7901/DDP) were obtained from the BeNa Culture Collection (Beijing Be Na Chuanglian Biotechnology Research Institute, Beijing, China). SGC7901/DDP cells were cultured in RPMI-1640 containing 10% FBS, 100 *μ*g/mL streptomycin, 100 U/mL penicillin, and 1 *μ*g DDP (maintaining drug resistance). The cells were incubated at 37°C in a 5% CO_2_ atmosphere. For the follow-up experiments, the cells were entirely cultured in RPMI 1640 without DDP.

### 2.3. MTT Assays

We used an MTT assay to validate drug resistance in SGC7901/DDP cells and evaluate the effects of BBD. Briefly, cells (1 × 10^4^ cells/well) in a logarithmic growth phase were seeded in 96-well plates. The next day, we replaced the original medium with media containing different drugs: 5-FU (0–25600 *μ*M), DOX (0–256 *μ*M), and DDP (0–40 *μ*M) and various concentrations of DDP combined with 0.5 mg/mL of BBD solutions with RPMI-1640 complete medium. The cell drug resistance was validated, and the reversal effect of BBD on cell viability was evaluated. After incubation with the drug for 48 h, SGC7901/DDP cells were supplemented with 100 *μ*l culture medium containing (0.5 mg/ml) MTT and incubated for 4 h (37°C, 5% CO_2_). Cells were then suspended in 100 *μ*l DMSO. Relative cell viability (*A* value) was determined using a plate reader (Multiskan FC, Thermo Fisher Scientific) at an absorbance of 570 nm. We used SPSS 21.0 to calculate IC50 based on the drug resistance index (RI) = IC50 (SGC7901/DDP)/IC50 (SGC7901) and reversal fold (RF) = IC50 (DDP/DOX/5FU)/IC50 (DDP/DOX/5FU + 0.5 mg/mL BBD) was calculated.

### 2.4. DOX and Rho123 Staining

SGC7901/DDP cells were seeded at a density of 4.0 × 10^5^ in 6-well plates and cultured overnight. When the cells reached 50%∼60%, different concentrations of BBD (0, 0.25, 0.5, and 1.0 mg/mL) were added and incubated for 48 h. Next, the supernatant was discarded, and 1 mL PBS was added to wash the cells three times. We used 4% paraformaldehyde to fix the cells for 10 min and then washed the cells with PBS 3 times. DOX (5 *μ*M) or Rho123 (5 *μ*M) staining solution was added and incubated with the cells for 15 min, followed by three 1 mL PBS washes. The samples were then observed and photographed with an inverted fluorescence microscope (400x).

### 2.5. DAPI Staining

After cell intervention, 4% paraformaldehyde was added to fix the cells for 15 min. Next, 10% DAPI staining solution (in PBS) was added to each well and cultured in the darkness for 15 min. The cells were then washed by PBS 3 times and imaged with a fluorescence microscope (400x).

### 2.6. Apoptosis Assays

The apoptotic rate was determined by flow cytometry. The Annexin V-fluorescein isothiocyanate (FITC)/propidium iodide (PI) apoptosis detection kit (Nanjing KeyGene Biotech Co., Ltd. Nanjing, China) was used. SGC-7901/DDP cells in a logarithmic growth phase were plated in six-well plates at the density of 4.0 × 10^5^ cells per well and treated with different concentrations of BBD (0, 0.25, 0.5, and 1.0 mg/mL) for 48 h, and 1 × 10^6^ cells were collected and washed twice with cold PBS. Cells were resuspended in 500 *μ*l 1 × binding buffer and then incubated for 15 min at room temperature in the dark following 5 *μ*l Annexin V/FITC and 5 *μ*l PI additions. For each analysis, 10,000 events were recorded.

### 2.7. Cyto-ID Autophagy Detection

SGC-7901/DDP cells were treated with different concentrations of BBD for 48 h, and the cells were centrifuged. Then, the 1 × 10^6^ cells in the pellet were resuspended in 0.5 ml of freshly diluted Cyto-ID green detection reagent (1 *μ*L Cyto-ID green detection reagent mixed with RPMI-1640 medium to a final volume of 2 mL). After incubation for 30 min at 37°C in the dark, the cells were identified by flow cytometry.

### 2.8. Western Blot Analysis

Protein expressions of Bax, Bcl-2, caspase-3, cleaved caspase-3, GAPDH, LC3, p62, Beclin 1, PI3K, AKT, p-AKT mTOR, and p-mTOR were examined by western blot analysis. After treated with BBD as above, SGC-7901/DDP cell proteins were extracted by radioimmunoassay (RIPA) buffer with an inhibitor cocktail (Thermo Fisher Scientific, USA) centrifuged at 14,000 rpm for 20 min at 4°C. The final supernatants were harvested, and protein concentrations were determined by a BCA protein assay kit. Total protein (30 *μ*g) was electrophoresed on 10% SDS-PAGE and then transferred to a PVDF membrane. Following incubation with 5% skimmed milk for 1 h at room temperature, primary antibodies (1 : 1000) were diluted with TBST solution and incubated with the membranes overnight at 4°C, washed 3 times (TBST, 10 min), and then incubated with secondary antibodies (HRP-conjugated 1 : 5000) for 1 h. Finally, we used Image Lab (Bio-Rad Laboratories, Inc., Berkley, California, USA) to detect protein. Three independent experiments were performed to obtain representative data.

### 2.9. Statistical Analysis

All representative data were obtained from three independent experiments. Statistical analyses were performed using IBM SPSS Statistics 21 software. *P* values <0.05 were considered to be statistically significant.

## 3. Results

### 3.1. SGC7901/DDP Cell Line Exhibited Multidrug-Resistant Cells

We utilized the resistance index (RI) to evaluate the resistance of MDR cells to various anticancer drugs. The SGC-7901/DDP cells exhibit MDR (RIs > 1.5) to DDP, DOX, and 5-fluorouracil (5-FU) with a corresponding RI of 1.86, 1.501, and 47.70, respectively (Table 1).

### 3.2. BBD Reversed the MDR of the SGC-7901/DDP

The ability of BBD to reverse the resistance of SGC-7901/DDP cells to several chemotherapeutic drugs is shown in Table 2. The reversal effect was evaluated by reversal fold (RF), with an RF > 1.5 indicating enhanced drug sensitivity. BBD (0.5 mg/mL) improved the sensitivity of SGC-7901/DDP cells to DDP, DOX, and 5-FU by 1.55-fold, 4.68-fold, and 3.56-fold, respectively. The results indicated that BBD significantly increased the cytotoxicity of anticancer drugs in SGC7901/DDP cells (Table 2).

### 3.3. BBD Downregulated ABCB1, ABCC1, and ABCG2 Expression and Increased the Accumulation of DOX and Rho123 in Gastric Cancer-Resistant Cells

**Figure 1:**
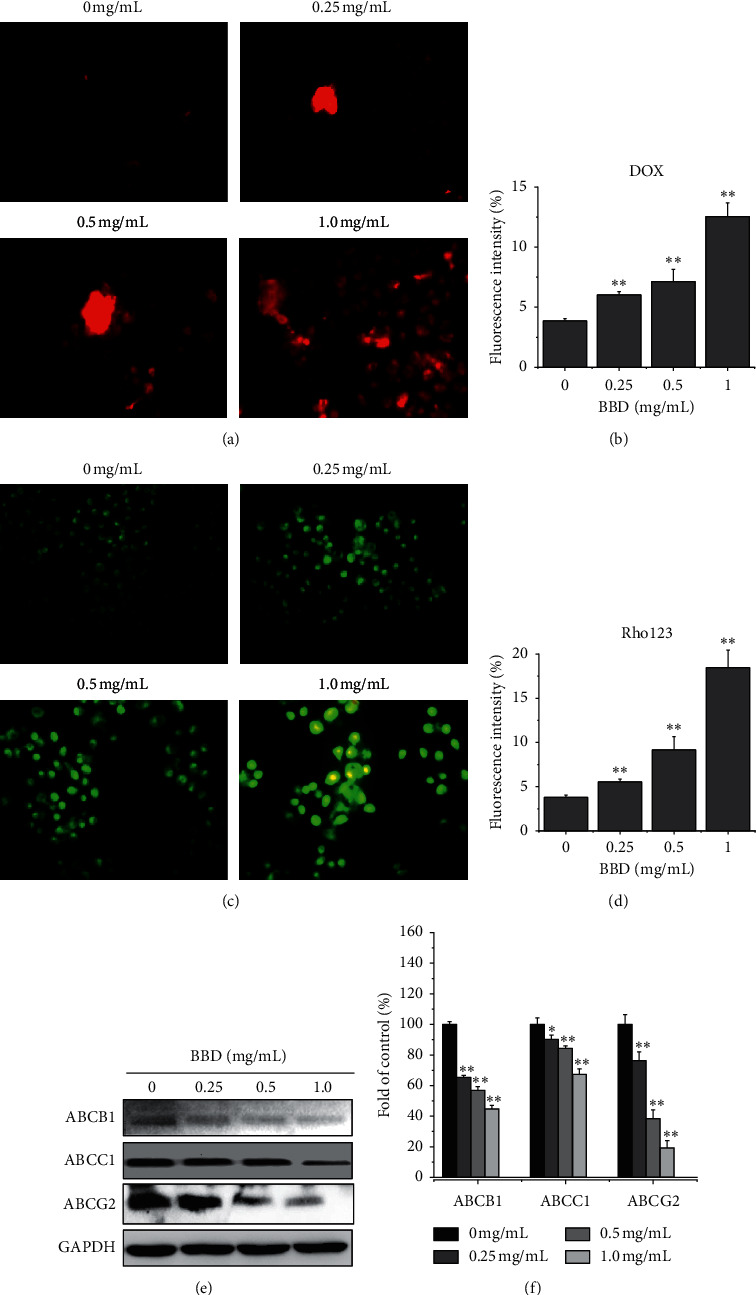
BBD increased doxorubicin and Rho123 accumulation and downregulated the protein expression of ABCB1, ABCC1, and ABCG2 in gastric cancer-resistant cells. SGC7901/DDP cells were treated with BBD (0.25, 0.5, or 1.0 mg/mL) for 48 h, doxorubicin and Rho123 staining were observed via fluorescence microscope ((a) and (c)), and the average fluorescence intensity of all the cells was calculated in five random fields ((b) and (d)). Expression and quantitative analyses of ABCB1, ABCC1, and ABCG2 protein are shown ((e) and (f)). ^*∗*^*P* < 0.05 and ^*∗∗*^*P* < 0.01.

The accumulation and efflux of chemotherapeutic drugs from tumor cells into the surrounding tissue in cells are important indicators for assessing MDR reversal. Our findings showed that 0.25, 0.5 and 1.0 mg/mL BBD increased the accumulation values of DOX to 6.03 ± 0.28, 7.13 ± 1.03, and 12.55 ± 1.15, over the levels in control cells (3.86 ± 0.19), respectively (*P* < 0.05) (Figures 1(a) and 1(b)), and the accumulation values of Rho123 were 5.4 ± 0.12, 14.7 ± 1.21, and 21.5 ± 1.78, over control cells (2.1 ± 0.15), respectively (*P* < 0.05) (Figures 1(c) and 1(d)). ABC family protein accounts for the accumulation of drugs. Western blot results showed that treatment with BBD (0.25, 0.5, or 1.0 mg/mL) significantly reduced the expressions of ABCB1, ABCC1, and ABCG2 compared with the control group (*P* < 0.05). ABCB1 expression rates were (100.00 ± 1.86)%, (65.35 ± 1.27)%, (56.86 ± 2.42)%, and (44.83 ± 2.19)% (*P* < 0.01); ABCC1 expression rates were (100.00 ± 4.19)%, (90.16 ± 2.93)%, (84.38 ± 1.52)%, and (67.25 ± 3.73)% (*P* < 0.05); ABCG2 expression rates (100.00 ± 6.46)%, (76.35 ± 5.63)%, (38.44 ± 5.76)%, and (19.36 ± 4.65)% (*P* < 0.01), respectively (Figures 1(e) and 1(f)).

### 3.4. BBD Induced Apoptosis of SGC7901/DDP Cells

**Figure 2:**
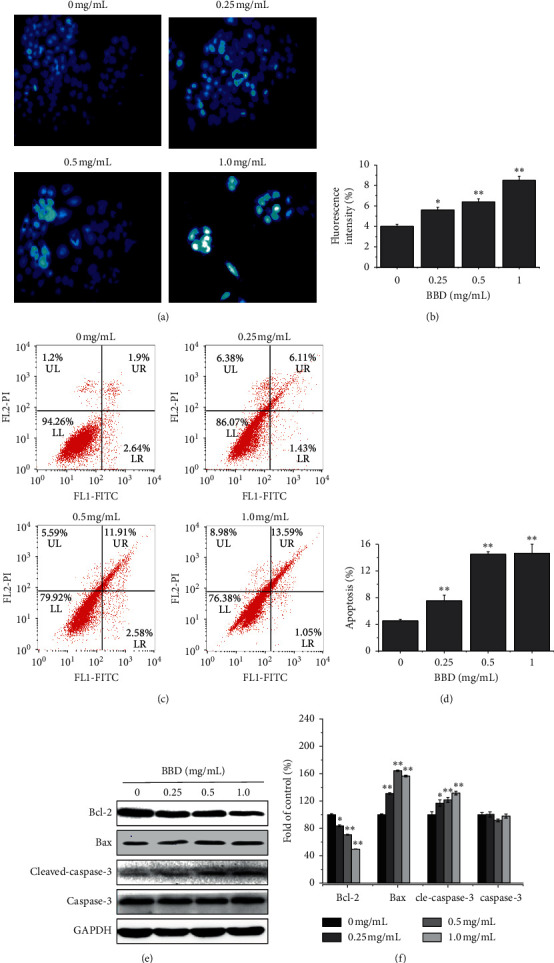
BBD induces apoptosis of SGC7901/DDP cells. SGC7901/DDP cells were treated with BBD (0.25, 0.5, and 1.0 mg/mL) for 48 h, and fluorescence microscopy was used to observe DAPI nuclear staining (a), and the average fluorescence intensity of all nuclei was calculated in five random fields (b). Annexin V/PI staining was used in flow cytometry to assess the proapoptotic effects of BBD (c). Data from apoptotic cell quantification (d). Cleaved caspase-3, caspase-3, Bcl-2, and Bax expression levels were detected by western blot (e). The quantification of each protein is shown (f). ^*∗*^*P* < 0.05, ^*∗∗*^*P* < 0.01 versus controls.

Resistance to apoptosis is one of the most important factors that contribute to MDR. As such, apoptotic induction may be a strategy to overcome MDR. The proapoptotic activity of BBD in SGC7901/DDP cells was tested by assessing DAPI and Annexin V/PI staining. DAPI staining showed that BBD could induce punctate apoptotic body formation, as shown in Figures 2(a) and 2(b). In cells treated with varying concentrations of BBD (0, 0.25, 0.5, and 1.0 mg/mL), Annexin V/PI staining was assessed via flow cytometry. The findings showed that the percentage of apoptotic cells was (4.54 ± 0.24)%, (7.54 ± 0.84)%, (14.49 ± 0.39)%, and (14.64 ± 1.36)%, respectively. Apoptosis was significantly elevated (*P* < 0.01), as shown in Figures 2(c) and 2(d). We also found that BBD regulated the expression of apoptosis-related proteins (Figure 2(e)). The expression levels of the Bcl-2 expression rate were (100 ± 1.34)%, (83.70 ± 1.33)%, (70.76 ± 0.76)%, and (49.56 ± 0.12)%, respectively (*P* < 0.05); the expression rates of Bax were (100 ± 1.21)%, (131 ± 1.17)%, (164.33 ± 1.05)%, and (156.53 ± 1.30)%, respectively (*P* < 0.01); the expression rates of cleaved caspase-3 were (100 ± 4.41)%, (116.82 ± 4.73)%, (121.63 ± 3.64)%, and (131.66 ± 2.65)%, respectively (*P* < 0.05); the expression rates of caspase-3 were (100 ± 3.12)%, (100.32 ± 3.69)%, (91.53 ± 1.96)%, and (98.04 ± 2.82)% respectively (*P* < 0.05) (Figure 2(f)). The above results suggest that BBD overcomes MDR in SGC-7901/DDP cells via apoptotic induction.

### 3.5. BBD Promoted Autophagy in SGC-7901/DDP Cells

**Figure 3:**
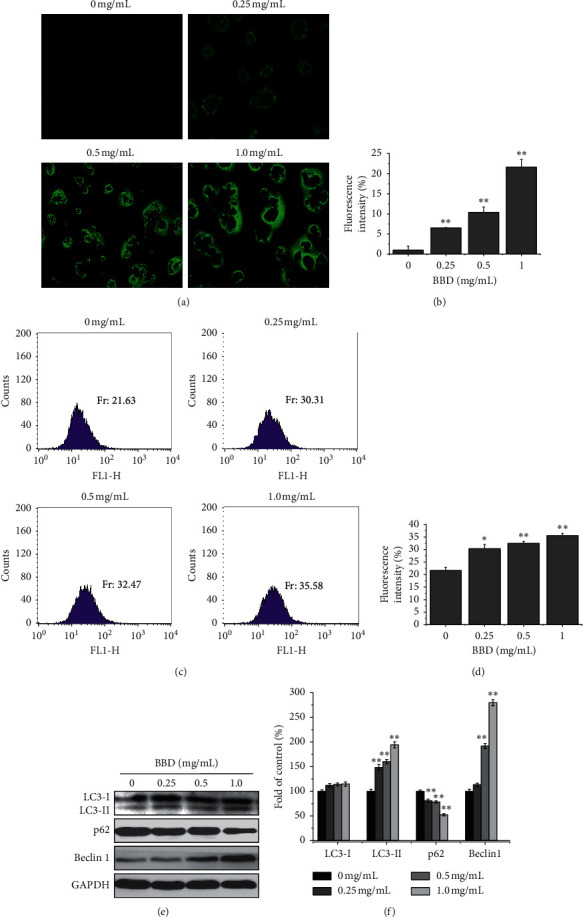
BBD promotes autophagy in SGC-7901/DDP cells. Fluorescence microscopy and flow cytometry were performed to detect autophagic activity via Cyto-ID staining of SGC-7901/DDP cells ((a) and (c)). The average fluorescence intensity of cells undergoing autophagy is shown in (b) and (d). LC3I, LC3II, p-62, and beclin 1 expression levels were detected by western blot (e). The quantification of each protein is shown in (f). ^*∗*^*P* < 0.05 and ^*∗∗*^*P* < 0.01.

**Figure 4:**
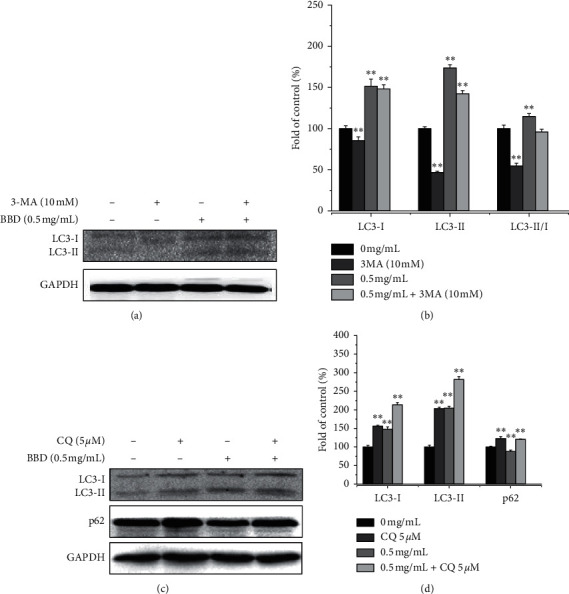
3-MA and CQ were used to further confirm the role of autophagy in SGC-7901/DDP cells. Expression and quantitative analysis of LC3I, LCII, and LC3II/I, and protein expression in SGC701/DDP cells treated with 3-MA (10 mM) ((a) and (b)). Expression and quantitative analysis of LC3I, LC3II, and p-62 protein in SGC701/DDP cells treated with CQ (5 *μ*M) ((c) and (d)). ^*∗*^*P* < 0.05 and ^*∗∗*^*P* < 0.01 versus controls.

To explore whether autophagy contributed to cell death, we assessed BBD treatment effects on autophagy in SGC-7901/DDP cells. Fluorescence microscope observation showed that the formation of green autophagy vacuoles and the fluorescence intensity were (1.0 ± 0.99)%, (6.5 ± 0.127)%, (10.4 ± 1.31)%, and (21.60 ± 1.92)% (*P* < 0.01), as shown in Figures 3(a) and 3(b). Flow cytometry was further used to detect the fluorescence intensity of gastric cancer drug-resistant cells after Cyto-ID staining, and the fluorescence intensity values were (21.63 ± 1.28)%, (30.31 ± 1.66)%, (32.47 ± 0.75)%, and (35.58 ± 0.84)% (*P* < 0.05) (Figures 3(c) and 3(d)). The Cyto-ID staining results showed that BBD promoted autophagy in SGC-7901/DDP cells. Moreover, we assessed autophagy-related protein expression and found that BBD increases the protein levels of LC3-II and Beclin 1 but decreased the expression of p62 compared with control cells (Figures 3(e) and 3(f)). Meanwhile, we used 3-MA and CQ to further confirm the role of autophagy in SGC-7901/DDP cells. 3-MA (an early inhibitor of autophagy) of 10 mM blocked the expression of LC3-II (Figures 4(a) and 4(b)), while CQ (a late inhibitor of autophagy) of 5 *μ*M upregulated the expression of LC3-II and p62. BBD upregulated LC3II, downregulated p62 expression, and promoted autophagy in drug-resistant gastric cancer cells (Figures 4(c) and 4(d)). Based on these results, we concluded that BBD promotes SGC-7901/DDP cell autophagy.

### 3.6. BBD Promoted Autophagy-Induced Apoptosis in SGC-7901/DDP Cells by Inhibiting the PI3K/AKT/mTOR Pathway

**Figure 5:**
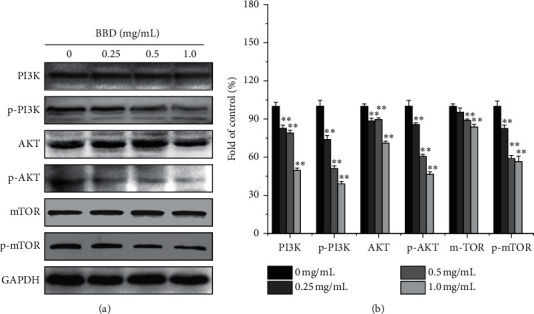
BBD promotes apoptosis and autophagy via blocking the PI3K/AKT/mTOR pathway in SGC-7901/DDP cells. Expression of PI3K, p-PI3K, AKT, p-AKT, mTOR, and p-mTOR proteins (a). Quantitative analysis of PI3K, p-PI3K, AKT, p-AKT, mTOR, and p-mTOR (b). ^*∗∗*^*P* < 0.01 versus controls.

**Figure 6:**
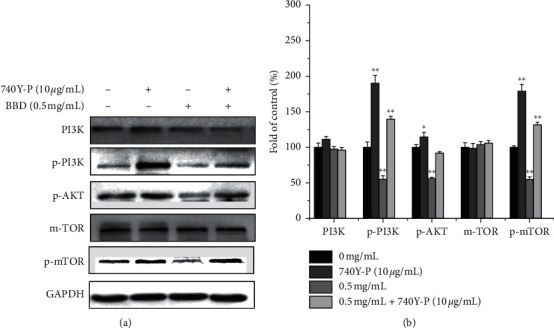
740Y-P significantly increased PI3K/AKT/mTOR activity. Expression of PI3K/AKT/mTOR pathway proteins in SGC701/DDP cells treated with BBD or 740Y-P (a). Quantitative analysis of protein expression (b). ^*∗*^*P* < 0.05, ^*∗∗*^*P* < 0.01 versus controls.

**Figure 7:**
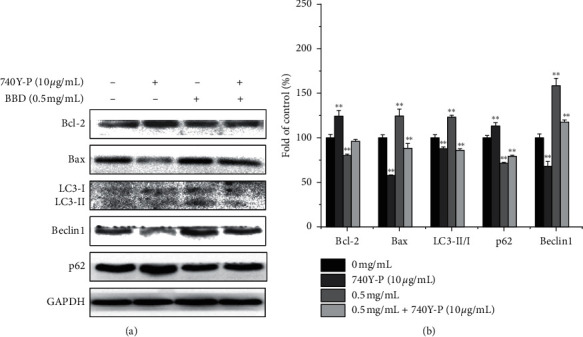
740Y-P was used to further verify that BBD triggers apoptosis and autophagy by inhibiting PI3K/AKT/mTOR pathway. Bcl-2, Bax, LC3II/I, p-62, and Beclin 1 expression levels were detected by western blot (a). Quantitative analysis of protein expression (b). ^*∗*^*P* < 0.05, ^*∗∗*^*P* < 0.01 versus controls.

Abnormal activation of PI3K/AKT/mTOR signaling is an important mechanism for promoting cancer biology. We examined whether BBD affects the PI3K/AKT/mTOR signaling pathway as a mechanism for its effects on the MDR of GC. Our results showed that BBD reduced p-PI3K, p-AKT, and p-mTOR expression and inhibited the PI3K/AKT/mTOR signaling (Figure 5). In addition, PI3K/AKT/mTOR is a common signaling pathway that regulates autophagy and apoptosis. To determine whether BBD regulated autophagy-induced apoptosis through the PI3K/AKT/mTOR signaling, we applied 740Y-P (an activator of PI3K) to the cells. As expected, 740Y-P inhibited autophagy and apoptosis. These results confirmed that BBD reversed MDR of GC via PI3K/AKT/mTOR signaling (Figures 6(a) and 6(b)). In addition, after 740Y-P and BBD intervention, apoptosis and autophagy-related protein results showed that 740Y-P inhibited apoptosis and autophagy in SGC7901/DDP cells, while BBD promoted apoptosis and autophagy in SGC7901/DDP cells (Figures 7(a) and 7(b)).

## 4. Discussion

With recent rapid developments in the Chinese economy, continuous improvement of people's living standards, and changes in the environment and diet, the incidence of gastric cancer has increased annually, which is a severe threat to human health. As a result, gastric cancer has become an undeniable social health problem. Over decades of medical research and therapeutic development, several chemotherapeutic regimens based on different anticancer drugs have improved the survival of many GC patients. However, chemotherapy in GC patients often fails due to the cancer cells' development of multidrug resistance (MDR) [17]. Therefore, increased emphasis has recently been placed on screening high efficiency and low toxicity drugs that can reverse or diminish tumor resistance. In the present study, we demonstrate that BBD may reverse MDR by downregulating PI3K/AKT/mTOR signaling to induce autophagy and apoptosis in the human drug-resistant cell line SGC-7901/DDP.

BBD, a traditional Chinese medicine formula, has the characteristics of a multicomponent, multitarget therapy and is widely used in cancer treatment. Preliminary studies have reported that BBD has been widely used to treat patients with various cancers with noted prevention of complications [13–16]. Wang et al. found that BBD can inhibit cell growth by inducing autophagy through PI3K/AKT/mTOR signaling and enhancing the antitumor effect of cisplatin in non-small-cell lung cancer (NSCLC) cells [18]. Still, the regulatory mechanism of BBD in GC multidrug resistance has not been fully clarified. Therefore, in this study, we first detected the drug resistance of gastric cancer drug-resistant cell lines. It was found that SGC7901/DDP drug-resistant cell lines had multidrug resistance, and the drug resistance index was RIs > 1.5. To verify the effect of BBD on the multidrug resistance of gastric cancer, we used low concentrations of BBD (0.25, 0.5 mg/mL) combined with different concentrations of chemotherapy drugs (DDP, DOX, and 5-FU) to treat gastric cancer drug-resistant cells. Compared with DDP, DOX, and 5-FU alone, our results showed that the RF values of 0.25 and 0.5 mg/mL BBD on DDP, DOX, and 5-FU were greater than 1.5, indicating that BBD reversed the drug resistance of gastric cancer drug-resistant cells. To further clarify the mechanism of its reversal of drug resistance, we analyzed cell drug resistance, apoptosis, autophagy, and the regulation mechanism of SGC7901/DDP.

It is well known that ABC transport protein-mediated MDR is one of the crucial causes of tumor chemotherapy failure [19, 20]. Therefore, potential molecular targets or biomarkers must be sought to mitigate MDR. P-gp (MDR1/ABCB1) relies on ATP energy to pump hydrophobic drugs from cells, which reduces intracellular drug concentration and causes resistance in tumor cells [21]. The expression of ABCB1 protein has been shown to be elevated in chemotherapy-resistant gastric cancer tissue compared to chemotherapy-sensitive cancer [22]. MRP (ABCC1) can transfer a combination of GSH and related drugs out of cells, leading to drug resistance [23]. Chen et al. found that ABCC1 increased the protein and gene expression of ABCC1 in a cancer cell line and induced *in vitro* gastric cancer cell line (SGC-7901/AS) resistant to arsenic trioxide [23–25]. ABCG2 is involved in mediating MDR by reducing drug penetration and intestinal absorption [26]. Accordingly, inhibiting ABCG2 can reduce MDR phenotypes and improve chemotherapy outcomes. Recent literature demonstrates that MDR1, MRP, and P-gp protein levels in SGC-7901/DDP and SGC-7901/VCR cells are increased [27]. Therefore, MDR1, ABCC1, and ABCG2 expressions were first measured in SGC-7901/DDP cells. Our findings indicate that BBD treatment for 48 h significantly lowers ABCB1, ABCC1, and ABCG2 expressions in SGC-7901/DDP cells, consistent with previous reports.

Apoptosis is an important defense mechanism to eliminate malignant cells and prevent cancer progression. The primary function of many antitumor drugs is to induce tumor apoptosis through various apoptosis-related signaling pathways [28]. One of the important mechanisms for the development of MDR in cancer cells in response to chemotherapy is to inhibit or evade apoptosis [29, 30]. There are two primary pathways tumor cells are stimulated to undergo apoptosis, the death receptor pathway (exogenous), and the mitochondrial-dependent pathway (endogenous). The key regulatory factors of endogenous pathways are Bcl-2 family proteins, including antiapoptotic factor Bcl-2 and proapoptotic factor Bax [31]. Overexpression of Bcl-2 and underexpression of Bax reduce cancer cell sensitivity to chemotherapy drugs to avoid apoptosis. Liquiritin (LIQ) has been shown to inhibit drug-induced apoptosis by increasing Bax and reducing Bcl-2 in cisplatin (DDP)-resistant gastric cancer cells [32]. Of the caspases, caspase-3 is the one most associated with several apoptotic pathways [33]. Therefore, BBD has been shown to overcome drug resistance and induce apoptosis in SGC7901/DDP cells by increasing Bax and cleaved caspase-3 expression while reducing Bcl-2 expression.

Autophagy is another molecular mechanism that induces cell death. Consequently, autophagy plays a vital role in preventing tumor growth in drug discovery [34, 35]. Extensive evidence suggests that increasing autophagy may help reverse MDR [36, 37]. For example, Wei et al. found that LIQ can induce apoptosis and autophagy by arresting the cell cycle at G0/G1 phase and enhancing the proapoptotic effect of DDP on human gastric cancer SGC7901/DDP cells [32]. Kou et al. found that Berberine improves chemosensitivity to cisplatin by enhancing apoptosis and repressing PI3K/AKT/mTOR signaling in gastric cancer [38]. Recent studies have confirmed autophagy as a scavenger in the apoptosis-blocked signal pathway, making MDR tumors sensitive to apoptosis [39]. As shown in Figure 3(a), fluorescence labeling showed that BBD treatment severely disrupted cell morphology. Western blot analysis demonstrated that BBD treatment greatly induced autophagy-related signals of LC3II and Beclin 1 but suppressed p62, leading to autophagy in SGC7901/DDP cells (Figures 3(e) and 3(f))

To verify the effect of BBD on the early autophagy of gastric cancer drug-resistant cells, BBD and 3-MA (an early autophagy inhibitor) were used to treat SGC7901/DDP cells. The results showed that 3-MA downregulated the expression of LC3II and inhibited early autophagy in SGC7901/DDP cells. BBD upregulated LC3II expression and promoted early autophagy in SGC7901/DDP cells. With 3-MA and BBD combined, the expression of LC3II was not completely blocked by 3-MA, suggesting that BBD may promote early autophagy in SGC7901/DDP cells through other mechanisms. For example, Bcl-2 inhibits Beclin 1-dependent autophagy or mitochondrial autophagy. In order to verify the effect of BBD on the advanced autophagy of gastric cancer drug-resistant cells, CQ (a late autophagy inhibitor) and BBD were used to intervene in SGC7901/DDP cells. The experimental results showed the with CQ and BBD intervention, CQ promoted the expression of LC3II and p62, and p62 inhibited advanced autophagy in SGC7901/DDP cells. Meanwhile, BBD downregulated p62 and promoted advanced autophagy. After a combined treatment of CQ with BBD, there was no significant difference in the expression of p62 compared with the CQ group, indicating that advanced autophagy induced by BBD was blocked by CQ.

Activation of the PI3K/AKT/mTOR signaling pathway has been shown to have carcinogenic effects in gastric cancer, and its regulatory pathways are closely related to genetic variation, cell proliferation, migration, invasion, cell cycle, apoptosis, autophagy, angiogenesis, multidrug resistance, and cell viability [21, 40–47]. Guo et al. found that Ubenimex inhibited the phosphorylation and activation of the PI3K/AKT/mTOR pathway and downregulated membrane transporter (such as P-gp and MRP1) expression, resulting in the intracellular accumulation of 5-fluorouracil and oxaliplatin [48]. Chen et al. reported that proton pump inhibitors inhibited the V-ATPase/PI3K/AKT/mTOR/HIF-1 alpha signaling pathways and downregulated TSC1/2 and Rheb expression in gastric cancer cells to reverse multidrug resistance [49]. In addition, Wang et al. found Babaodan induced autophagy and inhibited cell growth through the PI3K/AKT/mTOR pathway and enhanced antitumor effects of cisplatin in NSCLC cells [18]. So, the PI3K/AKT/mTOR signaling pathway was investigated to further elucidate the mechanism of BBD in reversing the MDR of the gastric cancer cell. Surprisingly, we found that several phosphorylated products in this pathway, such as p-PI3K, p-AKT, and p-mTOR, were reduced by BBD (Figure 5).

To further verify the inhibitory effect of BBD on this pathway, we used the PI3K agonist (740Y-P). As shown in Figures 6(a) and 6(b), our results showed that 740Y-P could significantly upregulate the phosphorylation of PI3K/AKT/mTOR proteins in SGC7901/DDP cells and promote the activation of this pathway, while BBD inhibited PI3K/AKT/mTOR signaling. When 740Y-P was combined with BBD, the inhibitory effect of BBD on this signaling pathway was offset by 740Y-P. The results of the 740Y-P intervention were consistent with prior reports [50], which showed that BBD could inhibit PI3K/AKT/mTOR signaling pathway activation and reverse MDR of SGC7901/DDP cells.

In addition, we further explored the role of the PI3K/AKT/mTOR pathway in BBD modulation of apoptosis and autophagy. As shown in Figures 7(a) and 7(b), our results indicate that 740Y-P-mediated activation of PI3K had a catalytic effect on p-PI3K, p-AKT, and p-mTOR and increased Bcl-2 and p62 expressions. In addition, it reduced Bax, autophagy-associated LC3II/I ratio, and Beclin 1 expression and inhibited apoptosis and autophagy in SGC7901/DDP cells. BBD downregulated Bcl-2 and p62 expression and promoted the expression of Bax, LC3II, and Beclin 1, promoting apoptosis and autophagy in SGC7901/DDP cells. After a therapy of BBD combined with 740Y-P, BBD promoted the apoptosis and autophagy of SGC7901/DDP cells, which were weakened or canceled by 740Y-P. These results showed that BBD can reverse MDR in gastric cancer cells via inactivating PI3K/AKT/mTOR signaling to induce apoptosis and autophagy.

## 5. Conclusions

In conclusion, the results of the present study indicate that BBD may be a promising candidate for the treatment of MDR in gastric cancer. However, as our study was performed only *in vitro*, additional studies are needed in animal models or humans to further confirm these therapeutic effects of BBD in gastric cancer.

## Figures and Tables

**Table 1:** The SGC7901/DDP cell line exhibited multidrug resistance to DDP, DOX, and 5-fluorouracil (5-FU).

Cell line	IC50 of DDP (*μ*M)	IC50 of DOX (*μ*M)	IC50 of 5-FU (*μ*M)
SGC7901	0.714	1.074	6.139
SGC7901/DDP	1.319	1.612	292.8
Resistance index	1.86	1.501	47.70

Resistance index (RI) = IC50 (SGC7901/DDP)/IC50 (SGC7901). The RIs of DDP, DOX, and 5-fluorouracil (5-FU) were 1.86, 1.50, and 47.70, respectively (Table 1). Resistance index of BBD on SGC7901/DDP cells.

**Table 2:** BBD reversed MDR in SGC-7901/DDP cells.

Chemotherapy drugs	IC50 of DDP (*μ*M)	IC50 of DOX (*μ*M)	IC50 of 5-FU (*μ*M)
BBD (0 mg/mL)	0.251	1.329	85.264
BBD (0.5 mg/mL)	0.162	0.284	23.952
Reversal fold (RF)	1.549	4.680	3.560

Reversal fold (RF) = alone chemotherapy IC50/combination chemotherapy. The ability of BBD to reverse the resistance of SGC-7901/DDP cells to several chemotherapeutic drugs is shown in Table 2. Reverse effect of BBD on SGC7901/DDP cells.

## Data Availability

The original data used to support the findings of this study are available from the corresponding author upon request.
